# The functional role of the inferior parietal lobe in the dorsal and ventral stream dichotomy

**DOI:** 10.1016/j.neuropsychologia.2008.11.033

**Published:** 2009-05

**Authors:** Victoria Singh-Curry, Masud Husain

**Affiliations:** UCL Institute of Cognitive Neuroscience & UCL Institute of Neurology, United Kingdom

**Keywords:** Hemispatial neglect, Sustained attention, Salience, Oddball, Locus coeruleus

## Abstract

Current models of the visual pathways have difficulty incorporating the human inferior parietal lobe (IPL) into dorsal or ventral streams. Some recent proposals have attempted to integrate aspects of IPL function that were not hitherto dealt with well, such as differences between the left and right hemisphere and the role of the right IPL in responding to salient environmental events. However, we argue that these models also fail to capture adequately some important findings regarding the functions of the IPL. Here we critically appraise existing proposals regarding the functional architecture of the visual system, with special emphasis on the role of this region, particularly in the right hemisphere. We review evidence that shows the right IPL plays an important role in two different, but broadly complementary, aspects of attention: maintaining attentive control on current task goals *as well as* responding to salient new information or alerting stimuli in the environment. In our view, findings from functional imaging, electrophysiological and lesion studies are all consistent with the view that this region is part of a system that allows flexible *reconfiguration* of behaviour between these two alternative modes of operation. Damage to the right IPL leads to deficits in both maintaining attention and also responding to salient events, impairments that contribute to hemineglect, the classical syndrome that follows lesions of this region.

## 1 Introduction

The cortical processing of visual information has been segregated – functionally, anatomically and conceptually – into a dorsal and ventral stream. Originally, Ungerleider and Mishkin proposed that the dorsal stream, connecting the primary visual cortex to the posterior parietal cortex (PPC), is dedicated to the processing of *spatial* information: the ‘where’ pathway (Mishkin, Ungerleider, & Macko, 1983; Ungerleider & Mishkin, 1982). By contrast, the ventral stream, extending from occipital cortex to the inferior temporal lobe, was considered to mediate *object* identification: the ‘what’ pathway. However, subsequent evidence suggested that both streams manipulate information about the nature of objects and their locations in space, and the dichotomy was revised by the pioneering work of Milner and Goodale. According to their model, the dorsal stream is responsible for the visual control of action while the ventral pathway enables enduring perceptual representations of our surrounding world (Milner & Goodale, 1995).

In Milner and Goodale's view, the dorsal *vision-for-action* system operates in real time, computing the absolute metrics of the target and its position in egocentric coordinates for eye or limb movements (Goodale, Westwood, & Milner, 2004; Milner & Goodale, 1995). Thus the dorsal stream delivers information directly to the motor system for immediate reaching, grasping or eye movements. By contrast, the ventral stream is dedicated to *vision-for-perception* and may also have a role in movement planning based on memory of an object and its relationship to other items. While many aspects of this model capture important features of the functional architecture of the cortical visual system, there is a sense of unease about how well the model accommodates all findings, as Milner and Goodale themselves acknowledge (see their recent defence, Milner & Goodale, 2008).

Many authors might agree broadly with their contention that the temporal lobe is specialized for perception while the superior parietal lobe (SPL) has a crucial role in the visual guidance of action. In our view, however, a crucial area of controversy is the proposed function of the human inferior parietal lobe (IPL). The disquiet here centres on whether this region fits easily into either of the proposed dorsal–ventral dichotomies, whether spatial versus object vision or action versus perception (Goodale et al., 2004; Milner & Goodale, 1995; Mishkin et al., 1983; Ungerleider & Mishkin, 1982). To their credit, right from the very beginning, Milner and Goodale made it clear that their model did not deal well with the human IPL. Initially, they speculated that it may have more ventral than dorsal stream functionality (Milner & Goodale, 1995). Later, Milner considered it to be ‘a nexus’ between the dorsal and ventral systems (Milner, 1997a). In fact, recent functional imaging studies, using either retinotopic mapping to demarcate visual areas (Sereno, personal communication) or simply getting observers to watch a movie (Sereno & Huang, 2006), have consistently failed to activate the IPL, suggesting this region may have functions that are distinctly different from other visual areas.

Here, we are going to argue that the human IPL simply does not fit the dorsal–ventral visual stream dichotomy. Although it may play a role in integrating information from these streams (Milner, 1997a), the IPL appears also to have other functions. Once this is acknowledged, it becomes easier to reconsider the extant data – without the baggage of any pre-existing theoretical framework – and ask what the evidence suggests this region is specialized for. To anticipate, we will argue that existing findings reveal considerable differences between the IPL in the two hemispheres of humans. The right IPL is involved in the detection of salient new events in the environment (Clark, Fannon, Lai, Benson, & Bauer, 2000; Gur et al., 2007; Huang et al., 2005; Kiehl, Laurens, Duty, Forster, & Liddle, 2001; Kiehl et al., 2005; Lagopoulos, Gordon, & Ward, 2006; Linden et al., 1999; Marois, Leung, & Gore, 2000; Williams et al., 2007) and also in sustaining attention on task goals (Adler et al., 2001; Hager et al., 1998; Johannsen et al., 1997; Pardo, Fox, & Raichle, 1991; Sturm et al., 1999, 2004; Vandenberghe, Gitelman, Parrish, & Mesulam, 2001), even in situations that do not require visual guidance of action or spatial shifts of attention. Lesions of this region also often lead to the syndrome of hemineglect (Heilman & Watson, 2001; Mort et al., 2003; Vallar & Perani, 1986).

By contrast, the left IPL in humans is specialized for functions underpinning limb praxis which have yet to be fully characterized (Buxbaum, Kyle, Grossman, & Coslett, 2007; Buxbaum, Kyle, & Menon, 2005; De Renzi, Motti, & Nichelli, 1980; Goldenburg, 1996; Haaland, Harrington, & Knight, 2000; Halsband et al., 2001; Pazzaglia, Smania, Corato, & Aglioti, 2008). In this review, we focus on the right IPL (although some of the findings regarding functions of the left IPL also clearly do not fit well either of the existing dorsal–ventral dichotomies).

We propose that a primary function of the right IPL is in *maintaining* attention on *current* task goals as well as encoding *salient events* in the environment so that task-sets can be speedily *reconfigured* to deal with new challenges. These aspects of control, traditionally considered solely to be the remit of frontal structures, are crucial for maintaining focus on a task in the face of distraction, and conversely also for flexibly switching to new external demands should that be necessary for optimal guidance of behaviour.

We argue that the right IPL is a crucial node in a fronto-parietal system which has often been associated independently by various authors with sustaining attention, detecting salient or novel events, phasic alerting and switching between task-sets. In our view, these behaviours are all different aspects of a cognitive system dedicated to allocating resources optimally, either to current behavioural goals or reconfiguring goals to novel, salient challenges in the environment. While many authors have viewed such processes as part of ‘executive control’ over behaviour, we do not find this term altogether helpful and prefer to specify the processes in terms of experimental task requirements.

We begin though by considering some aspects of the Milner and Goodale model, before examining two alternative schemes proposed by Rizzolatti and Matelli (2003) and Corbetta and Shulman (2002). In all these discussions we focus specifically on the proposed functions of the IPL. Next, we consider data from functional imaging, evoked potential and lesion studies that are currently not incorporated well by any of these models. Finally, we suggest a scheme of our own that takes into account these findings and attempts to integrate them into a coherent proposal regarding the contribution of the right IPL to behaviour.

## 2 The position of the IPL in Milner and Goodale's dorsal–ventral dichotomy

Much of the supporting evidence for Milner and Goodale's perception–action model comes from double dissociations between two pathological deficits of visual function: optic ataxia and visual agnosia. Optic ataxia refers to the condition in which patients experience difficulty in accurately reaching towards visual targets (Jeannerod, 1986; Perenin & Vighetto, 1988), but suffer no problems in correctly identifying such objects. In contrast, visual agnosia is characterised by a deficit in object recognition with intact visual control of actions (Milner, 1997b; Milner et al., 1991). Optic ataxia usually occurs following lesions of superior parietal areas (within the dorsal stream) (Auerbach & Alexander, 1981; Buxbaum & Coslett, 1998; Jeannerod, Decety, & Michel, 1994; Perenin & Vighetto, 1988), while visual agnosia is associated with temporal lesions (in the ventral stream) (Farah, 1995; Milner, 1995). This evidence has not gone without criticism, however (Pisella, Binkofski, Lasek, Toni, & Rossetti, 2006), although Milner and Goodale have countered some of these objections in their recent defence (Milner & Goodale, 2008).

The original anatomical studies leading to the exposition of two segregated cortical pathways were all performed on the monkey brain (Distler, Boussaoud, Desimone, & Ungerleider, 1993; Mishkin et al., 1983; Ungerleider & Desimone, 1986; Ungerleider & Mishkin, 1982). The ventral pathway projects from the striate cortex to the inferior temporal lobe, while the dorsal pathway terminates in the PPC which is divided into the SPL and IPL, respectively by the intraparietal sulcus (IPS). In the monkey, the dorsal pathway is considered to extend to the IPL. However, Milner and Goodale proposed that in humans, the dorsal stream terminates in the SPL and IPS, and does not project as far as the IPL. Such a view would be consistent with Brodmann's scheme (based on cytoarchitechtonic observations) that the human superior parietal region contains the homologue of the monkey IPL (Brodmann, 1909). But this leaves the human IPL unaccounted for in terms of the original dorsal–ventral dichotomy (Husain & Nachev, 2006).

**Fig. 1:**
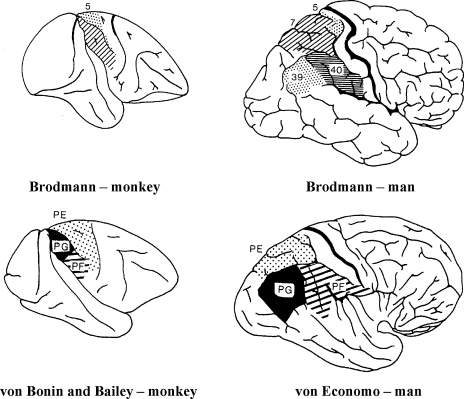
Anatomy of monkey and human posterior parietal cortex. According to Brodmann's examination of the monkey and human posterior parietal cortex, there is no monkey homologue of the human IPL. In contrast, von Bonin and Bailey's parcellation of the monkey posterior parietal cortex corresponds closely to that of the human as outlined by von Economo – here the monkey SPL and IPL are homologous to the human SPL and IPL. Reproduced from Husain (1991) in: Vision and Visual Dysfunction: Vol 13. Dyslexia, published 1991, [MacMillan Press Scientific & Medical] with permission of Palgrave MacMillan.

Homology between the human and monkey PPC has been a controversial issue. Von Bonin and Bailey (1947) drew parallels between the SPL and IPL in both species (Fig. 1). Their parcellation of the monkey PPC closely corresponds to that of the human, according to von Economo's analysis (Von Economo, 1929). This has led more recent investigators such as Rizzolatti and Matelli to suggest that homology between monkey *IPL* and human *SPL* would imply a jump of the IPL across the IPS during the course of evolution, which they consider to be highly unlikely (Rizzolatti & Matelli, 2003). Instead, they draw parallels between the SPL in humans and monkeys, and the IPL across both species. In their view, these regions are largely homologous. We discuss the issue of homology of parietal sub-regions more fully later, when we examine Rizzolatti and Matelli's scheme for the processing of visual information.

Milner and Goodale speculated that the *human* IPL may be a high-level spatial representational system which subserves perceptual awareness by transforming information derived from both streams, but *predominantly* the ventral stream (Milner & Goodale, 1995). This hypothesis is consistent with some of the object-related phenomenology that has been associated with hemineglect, the syndrome that often follows lesions of the IPL and temporoparietal junction (TPJ), particularly in the right hemisphere (Heilman & Watson, 2001; Mort et al., 2003; Vallar & Perani, 1986).

Although most investigators now consider hemineglect to be a multi-faceted disorder, with several potential components (Buxbaum et al., 2004; Husain & Rorden, 2003; Robertson, 2001; Samuelsson, Hjelmquist, Jensen, Ekholm, & Blomstrand, 1998), the most obvious problem in many patients with the syndrome consists of an inability to attend to events occurring to the contralesional side of space. Milner and Goodale's theory of IPL function (Milner & Goodale, 1995) gives a good account of ‘object-centred neglect’, where patients may fail to attend to the left *side of objects*, regardless of their location in space. This phenomenon is quite rare, however. By contrast, Milner and Goodale do not offer explanations for potentially more common spatial deficits in neglect: often conceptualised as impairments of egocentric spatial representation, directing attention or planning movements (Bisiach, 1993; Heilman, 1992; Heilman, Valenstein, & Watson, 2000; Kerkhoff, 2001; Mesulam, 1999).^1^ Aside from this, it is becoming increasingly apparent that *non-spatial* cognitive processes may also contribute to the neglect syndrome, e.g., the ability to sustain attention or encode stimulus salience (Husain & Rorden, 2003; Nachev & Husain, 2006; Robertson, 2001). We consider these processes in more detail later in our own reformulation of the function of this region but the key point here is that these processes have no manifestation in the Milner and Goodale scheme. The only attentional components they discuss in relation to their model are selective mechanisms: ‘…operating in the ventral stream to facilitate perceptual analysis of objects … alongside those in the dorsal stream which facilitate particular actions directed at those objects’ (Milner & Goodale, 1995).

In summary, the deficits that follow right IPL lesions in humans – spatial and non-spatial – make it difficult to place within the Milner and Goodale dorsal–ventral model (Husain & Nachev, 2006). Similarly lesions of the left IPL in humans are associated with limb apraxia (a syndrome involving difficulty copying or producing gestures and movements to command) which is also not dealt with easily in their scheme (Ietswaart, Carey, Della Sala, & Dijkhuizen, 2001). These concerns about IPL function have played a key role in the development of alternative proposals, including the model developed by Rizzolatti and Matelli (Rizzolatti & Matelli, 2003).

## 3 Rizzolatti and Matelli's two dorsal stream model

Rizzolatti and Matelli propose, on the basis of anatomical and functional evidence, that the dorsal visual stream is in fact formed by two sub-systems (Rizzolatti & Matelli, 2003). They argue that a *dorso-dorsal* stream has the basic characteristics of Milner and Goodale's dorsal stream and includes the SPL. Thus they interpret the data on optic ataxia and imaging studies on visually guided reaching as being broadly consistent with an online system for action. Their major departure concerns the IPL which they envisage as part of a separate *ventro-dorsal* stream. In their view, this stream plays a fundamental role in *both* perception and action. Specifically they consider the right IPL in humans to play a role in both spatial perception and action, with damage to this region causing hemineglect; while the left IPL has a central role in action recognition, grasping and manipulation, with lesions here leading to limb apraxia.

Like the original anatomical studies leading to the segregation of the cortical visual system into separate pathways (Mishkin et al., 1983; Ungerleider & Mishkin, 1982), Rizzolatti and Matelli's model developed from studies of the macaque visual system. As alluded to earlier, their analysis suggests to them that the IPS is functionally homologous in both macaques and humans, so it can be considered to divide the parietal cortex of both species into functionally similar SPL and IPL regions. One problem with considering that there are direct homologies between *all* parts of macaque and human parietal cortex is the hemispheric asymmetry that is so clear in humans and is an important part of the Rizzolatti and Matelli model. A similar difference between left and right IPL regions has never been demonstrated in macaques. This would also explain why there is no good monkey model of neglect that encompasses the severity, duration and impact on everyday functions of the syndrome observed in humans (Husain & Nachev, 2006). Furthermore, to the best of our knowledge there is no report of limb apraxia, as observed in humans, following lesions of the macaque IPL.

A second issue is that there may be differences between how the human and monkey PPC is organised, quite apart from hemispheric asymmetries. Comparative studies show that the IPS and IPL are markedly expanded in humans compared to the macaque monkey – at a ratio at least twice that of the overall increase in the rest of the cortical surface – particularly the angular gyrus and TPJ (Orban, Van Essen, & Vanduffel, 2004). Functionally, there also seem to be differences in this region between the two species (Orban et al., 2006, 2004), for example regarding analysis of 3D-structure-from-motion (Vanduffel et al., 2002). In fact, on the basis of functional magnetic resonance imaging (fMRI) studies performed in both humans and monkeys, the human IPS has been shown to contain more functional regions than the monkey IPS (Orban et al., 2006, 2003; Vanduffel et al., 2002). The human IPS has been reported to have at least four motion sensitive areas: ventral IPS, parieto-occipital IPS, dorsal IPS medial and dorsal IPS anterior; whilst the (rhesus) monkey IPS contains only one motion sensitive region (VIP) (Orban et al., 2006). The expansion of the IPS and IPL in humans may represent the anatomical correlate of characteristically human attributes, such as tool use, which would rely on a more detailed analysis of visual information (Rushworth, Behrens, & Johansen-Berg, 2006).

A third problem with the Rizzolatti and Matelli scheme is that it has recently been claimed that the monkey IPL is not formed by just two areas (as previously thought), but by four: Opt, PG, PFG and PF (Rozzi et al., 2006). Each of these regions was found to display distinct sets of connections with visual, somatosensory, auditory and limbic areas; in addition to robust interconnections between themselves. This newer data suggests that Rizzolatti and Matelli's formulation may be too simplistic.

Nevertheless, Rizzolatti and Matelli do attempt to address some of the issues regarding the IPL which are not really dealt with very well by the Milner and Goodale model. For example, they discuss the syndrome of limb apraxia in the context of the known responses of neurones in the IPL and IPS to action perception and control in the macaque. They also briefly address the spatial aspects of the neglect syndrome occurring after right IPL damage in humans. However, their account does not offer an explanation as to why individuals with neglect frequently have impairments of cognitive processes which do not have spatial perceptual or action oriented components (Husain & Rorden, 2003; Nachev & Husain, 2006; Robertson, 2001). These components of the neglect syndrome, we believe, also need to be addressed if accounts of right IPL function are to be credible. One important move in this direction comes from a model articulated by Corbetta and Shulman for attention systems in the human brain (Corbetta & Shulman, 2002).

## 4 Corbetta and Shulman's goal-directed and stimulus-driven streams

In their proposal, Corbetta and Shulman focussed on fractionating pathways from parietal to frontal cortex for directed attention (Corbetta & Shulman, 2002). Their scheme therefore does not directly address the functional architecture from primary visual cortex to parietal or temporal cortex. In this respect it is not concerned with all the issues dealt with either by Ungerleider and Mishkin or Milner and Goodale. Nevertheless, it is an important model which challenges the way in which both SPL and IPL functions are viewed from a visual system perspective. Rather unfortunately though, Corbetta and Shulman also used a dorsal and ventral distinction in their terminology, which does not map on to the traditional anatomical divisions for the visual system. Their *dorsal fronto-parietal* network incorporates the SPL, IPS and dorsal frontal cortex including the frontal eye fields, while the *ventral fronto-parietal* network, lateralised to the right hemisphere, involves the temporoparietal junction (TPJ), IPL and ventral frontal cortex including middle and inferior frontal gyri. They are not specific about which visual areas provide afferents to these streams.

According to this model, the dorsal fronto-parietal network is involved in the goal-directed or top–down selection of stimuli and responses; while the ventral fronto-parietal network detects salient, behaviourally significant events occurring in the environment (Corbetta & Shulman, 2002). In this scheme, top–down control of attention refers to prior knowledge about where in space to attend or what object features (such as shape, colour or motion) to search for in relation to current task or goal demands – *perceptual set*. It can also refer to advance information regarding what response needs to be produced, or *motor set*.

Such goal-directed signals for the allocation of spatial attention are usually assessed by tasks which provide a directional, *endogenous* cue regarding the subsequent location of a target. Functional magnetic resonance imaging experiments have shown that, unlike occipital regions which respond only transiently to such cues, sustained activation is observed in the IPS and frontal eye fields (FEF) in response to endogenous cues (Corbetta, Kincade, Ollinger, McAvoy, & Shulman, 2000). Thus dorsal fronto-parietal regions are activated when subjects direct spatial attention endogenously. Other studies have also separated *preparatory* signals for attending to stimuli from simple visual analysis, detection or response to such stimuli, consistently observing activity in the SPL, IPS and the FEF (Hopfinger, Buonocore, & Mangun, 2000; Kastner, Pinsk, De Weerd, Desimone, & Ungerleider, 1999). Dorsal fronto-parietal regions are also active during different types of visual selection, for example selection of motion direction (Shulman et al., 1999). These same areas are active too during action selection, e.g., both eye movement and arm related activity have been reported in the FEF and IPS (Connolly, Goodale, DeSouza, Menon, & Vilis, 2000; Connolly, Goodale, Menon, & Munoz, 2002).

What about the proposed *ventral* fronto-parietal network? Corbetta and Shulman consider the TPJ, which lies at the border of the IPL, to be a crucial node for encoding salient, behaviourally significant events. Stimulus salience refers to the properties of a stimulus that make it stand out from the surrounding background. For example, a red flower in a field of green grass stands out and hence rapidly draws attention because of its difference in shape and colour in relation to the green blades of grass. Similarly, abrupt visual onsets may also capture attention ‘bottom–up’. The effects of such sudden, distinctive events may be examined by tasks incorporating an *exogenous* cue – a flashed stimulus – which facilitates response to a target at the cued location. Such effects occur across different stimulus sensory modalities (Santangelo, Van der Lubbe, Belardinelli, & Postma, 2006).

Corbetta and Shulman argue that the ventral fronto-parietal network, involving TPJ and ventral frontal cortex, performs a ‘circuit-breaking’ function, reorienting attention to sudden, behaviourally salient events. This network is strongly right lateralised and therefore may have direct implications for the pathophysiology of hemispatial neglect (Corbetta & Shulman, 2002). Unlike the dorsal fronto-parietal network, the ventral fronto-parietal network is not activated by generating or maintaining an attentional set, but is strongly engaged by target detection (Corbetta et al., 2000; Perry & Zeki, 2000). Furthermore, when targets occur at an unexpected location – and are therefore very salient – activation is further enhanced in this network and shows even more lateralisation to the right hemisphere (Arrington, Carr, Mayer, & Rao, 2000; Corbetta et al., 2000). Importantly, activation in this processing stream is also observed when infrequently occurring stimuli occur at locations not requiring a spatial shift of attention, for example at gaze fixation (Marois et al., 2000). Right TPJ and ventral frontal cortex are also activated regardless of the stimulus modality of change (Downar, Crawley, Mikulis, & Davis, 2000).

Corbetta and Shulman argue that the anatomy of the neglect syndrome corresponds more closely to their ventral system than the dorsal one (Corbetta & Shulman, 2002). In addition, because they propose that the salience detection system in the TPJ is right lateralised, their model would also be consistent with neglect being far more frequent following right hemisphere lesions. Lesions to the TPJ are associated with impaired orienting to invalidly cued stimuli in contralesional space (Friedrich, Egly, Rafal, & Beck, 1998), a function originally attributed to the SPL (Posner, Walker, Friedrich, & Rafal, 1984) but since revised by many of the original investigators. Finally, studies which show neglect may follow focal lesions of the right ventral frontal cortex (Damasio, Damasio, & Chang Chui, 1980; Husain & Kennard, 1996) would also be in accord with this proposal.

Corbetta and Shulman argue that neglect patients with IPL or ventral frontal lesions have deficits primarily in stimulus detection rather than in top–down, goal-directed orienting of attention. We argue that this distinction may be an oversimplification and does not capture fully the role of the IPL in salience detection. Moreover, deficits in the ability to sustain attention are also prominent in many patients with neglect (Buxbaum et al., 2004; Husain & Rorden, 2003; Robertson, 2001; Samuelsson et al., 1998; Wilson & Manly, 2003b). It might be argued that the ability to sustain attention on a task or goal is also a ‘top–down’ cognitive process, dependent on subjects holding the task or goal ‘set’ in mind for the duration of the task. In fact, it has been demonstrated that a patient with a localised lesion of the right TPJ was unable to inhibit saccades to salient but task-irrelevant distractors as well as goal-relevant distractors, suggesting that *both* processes were affected by this lesion (Butler, Gilchrist, Ludwig, Muir, & Harvey, 2006). As we will discuss in the next section, numerous functional imaging studies in normal human subjects have identified the IPL in tasks incorporating sustained attention. These findings, we believe, raise questions about a simple distinction between the validity of a dorsal fronto-parietal system specialised for top–down control of behaviour and a ventral system dedicated to detecting stimulus salience.

## 5 The role of the inferior parietal lobe in vigilance and sustaining attention

According to traditional theories, attention is broadly divided into two domains: a *selectivity* aspect and an *intensity* aspect (Posner & Boies, 1971). Some authors have distinguished between vigilance and sustained attention as being two extremes of a continuum within the intensity domain. Thus *vigilance* has been considered ‘a state of readiness to detect and respond to *small* changes occurring at random time intervals in the environment’ (Mackworth, 1957) and is studied primarily through long, tedious tasks – vigils – requiring individuals to continuously monitor the environment for rare events. Detection of an infrequent blip on a radar screen would be an example where vigilant attention is considered to be deployed. *Sustained attention* on the other hand has been invoked in situations where the flow of information is more rapid, requiring continuous active processing and monitoring (Leclerq, 2002). For example, an interpreter giving an ‘on-line’ translation of a speech would be considered to be actively sustaining attention to the words of the speaker. In our view, both ends of this intensity spectrum require holding current goal or task instructions in mind in order to monitor incoming information from the environment and produce (motor) outputs which satisfy the goal/task demands. In this sense both vigilance and sustained attention require processes which are often termed as being ‘top–down’ in current parlance.

There is now ample evidence for a right hemisphere bias in the control of these intensity aspects of attention, even in terms of simple reaction time measures, from both patient studies (Howes & Boller, 1975) and investigations in normal control subjects (Sturm, Reul, & Willmes, 1989). Within the right hemisphere, lesion studies have specifically identified the IPL and ventral frontal cortex as crucial regions either for sustaining attention or vigilance, for example in patients with tumour excisions (Rueckart & Grafman, 1998; Rueckert & Grafman, 1996; Wilkins, Shallice, & McCarthy, 1987). Remarkably, the results of functional imaging studies have been extremely consistent with these findings.

**Fig. 2:**
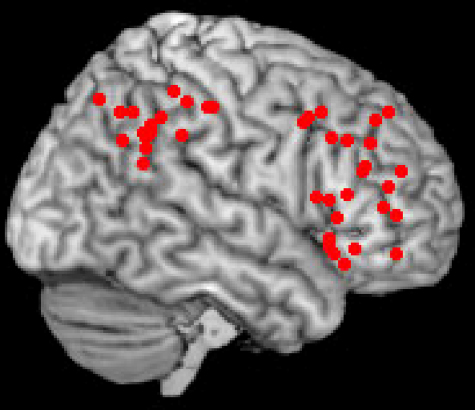
Activation sites associated with sustaining attention. Meta-analysis (performed in MRIcro–www.sph.sc.edu/comd/rorden/mricro) of sites of activation obtained during tasks assessing sustained attention in normal control subjects. Only areas within the right frontal and parietal lobes are shown here.

Thus while the SPL has been associated with spatial shifts of attention and the visual guidance of actions (Connolly, Andersen, & Goodale, 2003; Culham, Cavina-Pratesi, & Singhal, 2006; Vandenberghe et al., 2001), the IPL and ventral frontal cortex has been implicated repeatedly in tasks assessing sustained attention or vigilance in healthy subjects (Adler et al., 2001; Coull, Frackowiak, & Frith, 1998; Coull & Frith, 1998; Foucher, Otzenberger, & Gounot, 2004; Hager et al., 1998; Johannsen et al., 1997; Pardo et al., 1991; Paus et al., 1997; Sturm et al., 1999, 2004; Vandenberghe et al., 2001). Fig. 2 depicts the results of a meta-analysis we performed using MRICro software (www.sph.sc.edu/comd/rorden/mricro), of activations obtained in studies which used either positron emission tomography (PET) or fMRI (see Table 1 for full details). Investigations were included only if they employed a task in which subjects had to detect the occurrence of rare events at single locations (in various sensory modalities), or a version of the continuous performance task (CPT). The CPT typically involves the presentation of a relatively rapid, pseudorandom series of letters or digits at a rapid, fixed rate, with the instruction to respond to a particular stimulus letter or digit. Fig. 2 demonstrates that both vigilant and sustained attention protocols consistently activate the right IPL and ventral frontal cortex.

The role of the IPL and ventral frontal regions in sustaining attention is also supported by studies of patients with hemineglect. For example, it has been reported in a study of 44 right hemisphere stroke patients that individuals exhibiting neglect performed far worse on a non-spatial task of auditory sustained attention than control right hemisphere patients without neglect (Robertson et al., 1997). In fact performance on this task was found to be a better discriminatory test than line bisection, a more conventional measure of neglect. *Persistent* neglect has also been found in other studies to be related to an impairment in sustained attention (Hjaltason, Tegner, Tham, Levander, & Ericson, 1996; Samuelsson et al., 1998). Furthermore, it has been demonstrated that improving vigilance can ameliorate aspects of neglect (Wilson & Manly, 2003a). The use of computerised training tasks designed to increase endogenous maintenance of attention has been found not only to lead to improvements in tasks assessing neglect, but to greater activation in right hemisphere areas including preserved parts of the IPL (Sturm, Thimm, Kust, Karbe, & Fink, 2006; Thimm, Fink, Kust, Karbe, & Sturm, 2006). Finally, it has also been shown that the use of the noradrenergic agonist guanfacine can produce benefits in these patients, most likely by improving performance in maintaining attention (Malhotra, Parton, Greenwood, & Husain, 2006).

All these findings provide a strong evidence base for the role of the right IPL in maintaining attention, one of the intensity-based aspects of attention, which is not discussed in the models we have considered in this review. The right IPL also has a role in responding to salient events, as Corbetta and Shulman propose (Corbetta & Shulman, 2002). However, as we discuss, in the next section, their model may not fully capture the contributions of the region to this process.

## 6 The role of the inferior parietal lobe in salience detection and phasic alerting

Salience refers to the properties of a stimulus which make it stand out from the background. This may be because it represents something we have not encountered recently (*novelty*), or because its properties have behavioural significance to our current goal set (*behavioural* or *target-related salience*). Here, we propose also that a stimulus may be salient because it acts as a warning of an event of behavioural significance (*phasic alerting*), for example the ringing of an emergency alarm in a public building. Clearly these different types of salience have many similarities, but they also differ for example in the extent to which they involve ‘goal-directed’ versus ‘stimulus-driven’ processes. In other words, brain mechanisms involved in responding to salient stimuli need not be simply responding in a ‘bottom–up’ or exogenous fashion as suggested by the Corbetta and Shulman model (Corbetta & Shulman, 2002). We now discuss each of these categories of stimulus salience in turn and the role of the IPL in their mediation.

### 6.1 Target-related salience

Target-related salience refers to the process where the characteristics of a target stimulus must be held in mind to direct subsequent actions appropriately, depending on what is perceived in the environment or during the task. It is most frequently assessed using the *oddball paradigm*, which consists of infrequently occurring target stimuli (to which the subject must respond) embedded in a stream of frequently occurring standard non-target stimuli, to which responses must be withheld. In healthy subjects, event-related potentials (ERPs) have been frequently used to study the neurophysiological correlates of orienting to target stimuli in the oddball paradigm. Detection of such salient events leads to a characteristic positive response centred over the parietal lobe (Vaughan & Ritter, 1970) occurring approximately 300–500 ms after target presentation, but not after familiar non-targets. This wave is known as the P3 (Ritter, Vaughan, & Costa, 1968) or P300 (Smith, Donchin, Cohen, & Starr, 1970) response. Lesions of the TPJ lead to elimination of the P3 (Knight, Scabini, Woods, & Clayworth, 1989), whereas patients with prefrontal lesions have alterations of P3 over posterior areas (Barcelo, Suwazono, & Knight, 2000). Moreover, patients with hemineglect also show reduction of the P3 amplitude (Lhermitte, Turell, LeBrigand, & Chain, 1985).

**Fig. 3:**
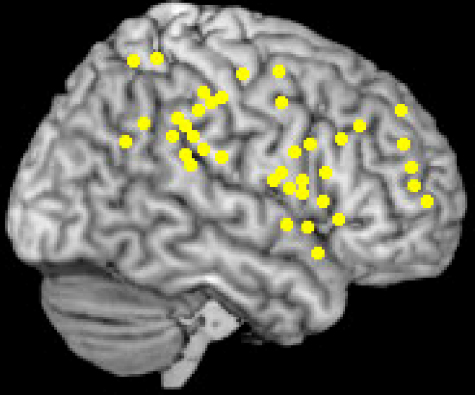
Activation sites associated with target related salience. Meta-analysis (performed in MRIcro–www.sph.sc.edu/comd/rorden/mricro) of brain activation sites associated with target detection during the oddball paradigm in healthy control subjects. Only right hemisphere frontal and parietal regions are shown.

In healthy control subjects, during target detection using this paradigm, the cortical areas most consistently activated on functional imaging are the right sided IPL, IPS, TPJ and frontal regions (Bunzeck & Duzel, 2006; Clark et al., 2000; Foucher et al., 2004; Gur et al., 2007; Huang et al., 2005; Kiehl et al., 2001, 2005; Lagopoulos et al., 2006; Linden et al., 1999; Marois et al., 2000; Williams et al., 2007). This is illustrated in Fig. 3, which plots right hemisphere activation foci obtained in a meta-analysis we performed of these studies (listed in Table 2). The investigations included in this analysis employed the oddball paradigm using stimuli of any sensory modality, but occurring at a single location only.

Performance on the oddball task clearly may involve ‘bottom–up’ or ‘stimulus-driven’ capture of attention by virtue of targets being rare, as Corbetta and Shulman argue (Corbetta & Shulman, 2002). However, in current terminology, keeping the target in mind during the oddball task might also be considered to be a ‘top–down’ or ‘goal-directed’ activity. In our view, therefore, the suggestion that neglect patients might have problems only in ‘bottom–up’ stimulus detection (Corbetta & Shulman, 2002) fails to capture all aspects of the contribution of the IPL. Our proposal is that the right IPL plays a key role in responding to salient targets which requires *both* the task goal to be maintained – so targets can be discriminated from non-targets – as well as detection of successive stimuli in the task.

### 6.2 Novelty

New events or objects, which have not been encountered in a particular behavioural context before are highly salient and also easily attract attention. This is an essential feature of a nervous system which encourages exploration of the surrounding environment. Like target-related salience, novelty has been studied using the oddball paradigm. In such tasks, in addition to infrequently occurring targets which require a response, there are occasional new stimuli which have not been presented previously. Subjects are instructed to respond only to the targets and are usually not given any instructions about the novel stimuli. Like targets, novel stimuli elicit a P3 ERP response centred over parietal cortex, even when no response to these items is required. But the positive wave occurs slightly earlier (sometimes referred to as P3a) than that which occurs to targets (P3b) (Courchesne, Hillyard, & Galambos, 1975; Squires, Squires, & Hillyard, 1975). Lesions of the TPJ lead to abolition of both the P3a and P3b (Knight et al., 1989).

**Fig. 4:**
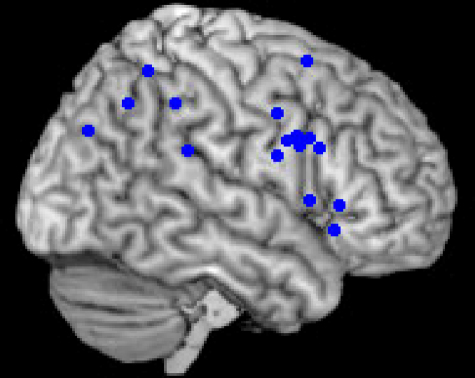
Activation sites associated with novelty detection. Meta-analysis (performed in MRIcro – www.sph.sc.edu/comd/rorden/mricro) of brain regions associated with novelty detection during the oddball paradigm in control subjects. Only right hemisphere regions in the frontal and parietal lobes are demonstrated here.

While the areas of activation obtained during functional imaging studies in healthy subjects seem to occur more posteriorly in response to novelty than targets, they too predominantly involve the IPL, TPJ and ventral frontal lobe (Bunzeck & Duzel, 2006; Downar, Crawley, Mikulis, Davis, 2002; Gur et al., 2007; Kiehl et al., 2001, 2005). This is demonstrated in Fig. 4, which plots the results of a meta-analysis we performed of functional imaging studies (listed in Table 3) using the oddball paradigm to determine the anatomy of brain regions associated with the processing of stimulus novelty. Again, all of the investigations we included presented stimuli at a single location only, but a variety of sensory modalities was employed.

Again, as with orienting to target stimuli in the oddball paradigm, it might be argued that detection of novel events occurs in a primarily ‘bottom–up’ fashion; but clearly memory of previous items also needs to be maintained for a novel stimulus to be correctly judged as new. Thus, in our view, a ‘stimulus-driven’ account also fails to capture all aspects of the processes required to respond to novelty in such paradigms.

### 6.3 Phasic alerting

The final aspect of salience we discuss is phasic alerting, a process that has usually not been considered in this context but considered separately, under the intensity aspects of attention. Phasic alerting refers to a readiness to detect or respond to environmental changes occurring as a result of an exogenous warning stimulus (Posner & Boies, 1971), which may be in the same modality as the subsequent target stimulus or a different one. There may be predefined associations between an alerting stimulus and one which follows it, for example a cue presented a set interval before a visual target. Posner and Boies’ studies demonstrated that reaction times to targets following phasic alerting cues were least if the interval between cue and target was 500–1000 ms (Posner & Boies, 1971). On the other hand, there may not be any predetermined stimulus–stimulus or stimulus–response association, in which case the alerting cue becomes very similar to a novel one. In fact, in some respects, all salient stimuli are phasic alerting, to varying degrees. Here we will consider a phasic alerting stimulus to be one which warns the subject of an impending target, but is of no other informational value. It has of course been shown that an alerting cue which orients a subject to the spatial location of an impending target, activates the right IPS and TPJ (Corbetta et al., 2000; Kastner et al., 1999). However, it is important to note that there are also studies which suggest these regions are important in the detection of cues which provide no such predictive information (Fan, McCandliss, Fossella, Flombaum, & Posner, 2005; Thiel & Fink, 2007).

**Fig. 5:**
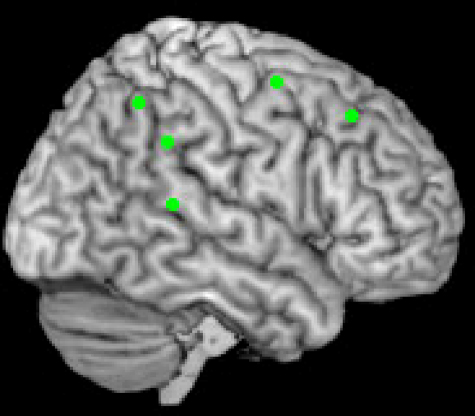
Activation sites associated with phasic alerting. Meta-analysis (performed in MRIcro–www.sph.sc.edu/comd/rorden/mricro) of regions activated by non-informative warning cues in healthy control subjects. Right hemisphere frontal and parietal regions only are demonstrated here.

In one such study (Thiel & Fink, 2007), a simple target detection paradigm was used in which some of the targets were preceded by a visual or auditory cue (variable cue-target interval, so as not to be temporally predictive). The other investigation (Fan et al., 2005) employed the attention network test (ANT) which is designed simultaneously to probe the effect of a non-informative cue (*alerting* condition), a spatially informative cue (*orienting* condition) and a condition in which the target arrow stimulus is flanked either by incongruent or congruent arrow stimuli (*conflict* situation, obtained by subtracting the effect of congruent from incongruent). Fig. 5 plots, in MRICro, the right fronto-parietal activations obtained in these two studies (which are the only ones we found in the literature to list coordinates of activation in response to a non-spatially informative alerting cue). Again, the IPL and TPJ are implicated. In the right frontal lobe, the activation centroids appear to be in the middle, rather than inferior, frontal gyrus.^2^

Lesions of the right hemisphere have long been known to impair alerting responses, measured with galvanic skin responses (Heilman, Schwartz, & Watson, 1978) or heart rate changes to warning cues (Yokoyama, Jennings, Ackles, Hood, & Boller, 1987). Conversely, patients with hemineglect following right hemisphere lesions benefit from an alerting tone during a task designed to assess the severity of their leftward inattention (Robertson, Mattingley, Rorden, & Driver, 1998). Posner and Petersen argued that ascending noradrenergic pathways from the locus coeruleus (LC) play a key role in alertness specifically through their innervation of right frontal and parietal regions (Posner & Petersen, 1990), with the parietal cortex in particular appearing to receive a dense projection (Foote & Morrison, 1987). They pointed to electrophysiological studies which suggested a crucial function of LC noradrenergic cells in arousal. For example, the activity of these neurones is reduced in states of low arousal (Aston-Jones, Gonzalez, & Doran, 2007).

Recently, however, our understanding of the role of the LC noradrenergic system has been revised to a more sophisticated formulation. Aston-Jones and colleagues argue that the LC contributes to the regulation of attention between a focussed, selective attention state (that facilitates responses to targets and filters out distractors) and a scanning, labile state that allows flexible responding to new events, i.e. to stimuli that are not targets but may nevertheless be important (Aston-Jones & Cohen, 2005; Aston-Jones, Iba, Clayton, Rajkowski, & Cohen, 2007). As we have discussed, there is evidence for involvement of the right IPL in both of these modes of operation: maintaining attention and responding to novel, salient events.

In summary, the evidence points to a role of the right IPL in phasic alerting, which may be a special case of a response to a salient stimulus in the environment that acts to reconfigure task goals, possibly via interactions involving a noradrenergic input from the LC.

## 7 The process of reconfiguration

How does reconfiguration occur? One way to examine this issue is to look at data on task switching. Functional imaging data and ERP evidence suggests a role for the IPL – as well as frontal regions – in task set reconfiguration, although not necessarily lateralized to the right hemisphere (Buchsbaum, Greer, Chang, & Berman, 2005; Rushworth, Passingham, & Nobre, 2005; Travers & West, 2008).

Tests assessing how we switch between two or more tasks involve the reconfiguration of a number of discrete processes (Wager, Jonides, & Reading, 2004). Task switching may involve a shift in the *rule* used to process stimuli in search of behavioural targets: for example from spatial location to object attributes of items. It may also involve a change in the *motor response*, e.g., which hand to respond with following, target stimuli. Unfortunately, most paradigms assessing task switching involve both of these processes, as well as differing degrees of working memory load, and a variety of bilateral frontal and parietal foci of activation are found in such studies. For this reason, meta-analysis may be particularly useful in elucidating the critical regions underlying reconfiguration.

One recent review undertook meta-analyses of neuroimaging studies of three types of paradigm: the Wisconsin Card-Sorting Task (WCST), task-switching studies and the go/no-go task, as well as a crucial conjunction analysis of all three paradigms (Buchsbaum et al., 2005). The WCST requires subjects to sort cards according to a rule which they must learn by trial and error. After a set number of trials, this rule changes and participants must ‘shift set’ in order to determine the new way in which they must sort the cards. This task was originally developed to probe human abstraction and ability to switch set. However, it clearly involves other cognitive processes, such as working memory and the ability to learn from positive and negative feedback. This is in contrast to ‘purer’ tests of task-switching in which an instructional cue specifies explicitly which of two rules should be used. Finally, in the go/no-go task, subjects are instructed either to respond (go) or not to respond (no-go) to a predefined set of stimuli embedded in a stream of rapidly presented items. The stimuli are presented such that the ‘go’ response predominates, so that when a ‘no-go’ stimulus occurs, the subject has to overcome a predisposed tendency to respond. This ability to inhibit a pre-potent, conflicting response is also a key component of both task switching and the WCST.

**Fig. 6:**
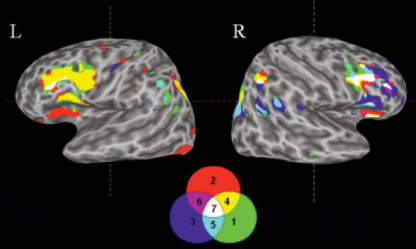
Conjunction analysis of studies using the WCST, task-switching and go/no-go paradigms. Three-dimensional surface rendered views of meta-analyses and all possible conjunctions of the following paradigms: 1 = WCST, 2 = task-switching, 3 = go/no-go task, 4 = WCST and task-switching, 5 = WCST and go/no-go, 6 = task-switching and go/no-go, 7 = WCST, task-switching and go/no-go. Reproduced from Buchsbaum et al. (2005) *Human Brain Mapping*, 25(1), 35–45. Copyright (2005 John Wiley & Sons). Reprinted with permission of John Wiley & Sons Inc.

The right IPL and ventral frontal cortex were identified as major foci of activation in all three meta-analyses, along with their left-sided counterparts. However, in a conjunction analysis of all three types of study, the right – and not the left – IPL and ventral frontal cortex were found to be substantially activated (Fig. 6). This finding suggests that a process common to all three of these paradigms – such as the ability to overcome conflict between a previous response and a new one – depends upon the right, rather than the left, IPL.

As with the process of reconfiguration, most studies investigating the effect of potentially conflicting responses, have focussed on the frontal lobes (Botvinick, Cohen, & Carter, 2004; Nachev, Rees, Parton, Kennard, & Husain, 2005; Rushworth, Buckley, Behrens, Walton, & Bannerman, 2007). It is, however, becoming clear that this crucial component of the reconfiguration process, is also associated with PPC activity (Liston, Matalon, Hare, Davidson, & Casey, 2006; Stoet & Snyder, 2007). A recent study performed with neglect patients also supports this contention (Coulthard, Nachev, & Husain, 2008). Coulthard and colleagues used a (vertical) directional flanker task, to demonstrate that patients with posterior parietal lesions show a paradoxical *facilitation* of rightward movements in the presence of conflicting leftward response plans. In contrast, neglect patients with frontal damage had increased costs of conflict for both leftward and rightward movements. The authors argue that the findings suggest the right PPC normally acts as a crucial stage for the automatic activation of competing motor plans, whilst frontal regions act to inhibit action plans which are not relevant to current task goals. Importantly, patients with left parietal lesions did not demonstrate a similar facilitation of leftward movements in the context of conflicting rightward response plans. This, like the conjunction meta-analysis of WCST, task-switching and go/no-go paradigms (Fig. 6), suggests that resolution of response conflict may be predominantly a function of right, rather than left, parietal cortex.

Neurophysiological evidence suggests that task-switching is accompanied by a parietal slow wave which appears to be a P300 or P3 response (Rushworth et al., 2005; Travers & West, 2008). As discussed earlier, detection of salient targets is also associated with a parietal P3 response (P3b), as is the detection of novel stimuli (denoted P3a). However, the P3a in response to novel stimuli occurs slightly earlier and more anteriorly (Herrmann & Knight, 2001) than the P3b evoked by task-relevant events. Moreover, the P3a is generally of smaller amplitude and/or of shorter latency than the P3b, with a greater rate of habituation, particularly over parietal regions (Comerchero & Polich, 1999; Courchesne et al., 1975; Katayama & Polich, 1998; Polich & Comerchero, 2003; Volpe et al., 2007; Yamaguchi & Knight, 1991).^3^ Thus there is a difference between the P3 response to salient *task-related* stimuli – the P3b – and to novel stimuli that may not be relevant to the task – the P3a.

Intriguingly, converging evidence from animal neurophysiological, pharmacological and lesion studies, as well as some human studies, suggests that the P3 recorded over cortical regions reflects phasic activity of the LC noradrenergic system, which sends dense projections to the parietal cortex (for review see Nieuwenhuis, Aston-Jones, & Cohen, 2005). For example, lesions of the LC in monkeys lead to abolition of P3-like cortical responses (Pineda, Foote, & Neville, 1989). Consistent with such findings, computational modelling by Dayan and Yu has suggested the possibility that phasic noradrenergic activity might act as a ‘neural interrupt signal’, re-setting or reconfiguring ongoing processing, leading to a shift in behaviour towards a task-engaged state (Dayan & Yu, 2006).

It has been generally acknowledged that noradrenergic LC cells fire en masse either phasically or tonically in response to afferent input (Aston-Jones & Cohen, 2005; Aston-Jones, Gonzalez, et al., 2007; Berridge & Waterhouse, 2003). Aston-Jones and colleagues have proposed that *phasic* noradrenergic activity facilitates focussed selective responding with effective filtering out of distractors. On the other hand, an increase in *tonic* LC activity (associated with reduced phasic activity) shifts behaviour into an exploratory, more distractible state (Aston-Jones & Cohen, 2005). The computational modelling work performed by Dayan and Yu extends this concept, suggesting that alterations in the tonic activity of LC noradrenergic activity signals unexpected events in the surrounding environment, for example, changes in the nature of a task or the behavioural context in which it is being performed (Dayan & Yu, 2006; Yu & Dayan, 2005). They envisage phasic activity (which correlates with the P3) to signal the occurrence of uncertain events *within* a task, alerting the subject to the occurrence of a goal relevant stimulus (such as a target or a predetermined signal to switch stimulus–response contingencies) and interrupting the default state (Dayan & Yu, 2006). In this way, phasic noradrenergic activity facilitates sustained and accurate performance of a task.

**Fig. 7:**
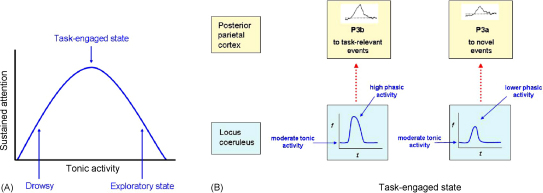
Postulated relationship between locus coeruleus activity, posterior parietal function and behavioural state. (A) The relationship between tonic levels of noradrenergic activity in the locus coeruleus (LC) and the ability to sustain attention on a task follows an inverted U-shaped function. Low levels of tonic activity are associated with drowsiness, moderate levels with efficient *task-engaged* behaviour and higher levels with a more distractible, *exploratory* state. (B) In the task-engaged state, LC tonic activity is moderate, with optimal phasic bursts in response to task-related targets (*f*, firing rate of LC neurons; *t*, time or latency). This leads to a *P3b* potential in the posterior parietal cortex, facilitating accurate task performance. *Novel* or *infrequently* occurring events (of no task-relevance) can also produce phasic LC responses. These are associated with a *P3a* potential in parietal cortex, which is generally smaller than the P3b potential. The P3a does not correlate with behavioural responses when performance is task-engaged. Note that, in contrast to either target or infrequent novel stimuli, *frequently* occurring task-irrelevant stimuli do not evoke P3 responses.

The relationship between tonic noradrenergic activity and function is thought to follow an inverted U-shaped curve (Fig. 7A), with an optimal level of focussed performance being associated with a moderate level of noradrenaline, while low noradrenergic levels are associated with drowsiness and high levels with distractibility (Aston-Jones & Cohen, 2005). Importantly, the level of tonic activity appears to influence the extent of *phasic* noradrenaline release. At low tonic levels, when the animal is drowsy, there is very little phasic activity, and similarly at very high tonic levels. But between these two extremes – at moderate tonic noradrenergic levels – phasic LC bursts are most effective and are strongly correlated with accurate target detection (Aston-Jones, Rajkowski, Kubiak, & Alexinsky, 1994) and, by inference, the P3b potential recorded over parietal cortex in response to salient task-related stimuli (Fig. 7B). It is in this condition that behaviour seems to be most easily maintained on task demands, corresponding to our view of the state of sustained attention in human observers.

Under these circumstances, we hypothesise that *novel* task-irrelevant stimuli also cause phasic bursts of activity within LC neurons, but of smaller amplitude or shorter duration. Studies in humans show that under such conditions the P3a recorded over cortical regions is lower amplitude and/or shorter latency (Yamaguchi & Knight, 1991). If baseline or tonic noradrenergic levels were to increase, then we envisage that responses to novel or distracting stimuli would become more prominent. Thus behaviour becomes more distractible and exploratory in nature and disengagement from the task occurs, accompanied by a reduction in LC phasic activity and parietal P3b potentials to targets (Aston-Jones & Cohen, 2005; Aston-Jones et al., 1994; Usher, Cohen, Servan-Schreiber, Rajkowski, & Aston-Jones, 1999).

In summary, we argue that *phasic* bursts of LC noradrenergic activity (on a background of moderate tonic levels) induce, via parietal regions, a goal-focussed task-engaged state, enhancing sustained attention to task demands and facilitating the detection of task-relevant events (indexed by the P3b). On the other hand, increases in LC *tonic* activity shift behaviour towards a more distractible and exploratory state, favouring responses to novel environmental stimuli. These are the two broadly complementary, aspects of attention – maintaining attentive control on current task goals and responding to salient new or alerting stimuli in the environment – which we consider to be a crucial aspect of right IPL function.

But what drives the LC noradrenergic input to the parietal cortex and what governs the interplay between phasic and tonic modes of functioning? The answers to these questions remain to be established. However, it may be important to note that in addition to receiving subcortical afferents, there are prominent cortical projections to the LC from medial frontal and orbitofrontal structures (Aston-Jones et al., 2002; Rajkowski, Lu, Zhu, Cohen, & Aston-Jones, 2000) which might play a key role in modulating its responses. For example, it has been demonstrated that the amplitude of LC phasic responses to targets on a signal detection task, is altered by the motivational significance (i.e. associated reward) of the stimulus (Aston-Jones et al., 1994; Rajkowski, Majczynski, Clayton, & Aston-Jones, 2004). Such *motivational salience* signals in the LC may come via frontal regions and act to modulate the noradrenergic innervation to parietal cortex. The PPC also receives direct connections from frontal regions (Schmahmann et al., 2007).

In fact, the PPC appears to be an important hub where several different types of information – sensory, motor, goal-related and reward – converge. Indeed, recent evidence demonstrates that the IPL is at the heart of a ‘structural core’ of the human cerebral cortex, as one of the most densely interconnected cortical regions (Hagmann et al., 2008). Such connectivity ideally places the IPL at the centre of a network where these different types of information may compete, with signals from the LC biasing the outcome of the competition depending upon whether the subject is in a task-engaged (sustained attention) or distractible, exploratory state.

## 8 The role of the right inferior parietal lobe in controlling behaviour

In the last few sections, we have discussed how the IPL plays a central role in networks which underlie both sustained attention and various forms of response to salient stimuli in the environment. Maintaining attention on current task goals is crucial for successful accomplishments but just as important is the ability to adapt to changing circumstances by reconfiguring task goals should the need arise, based on salient new information. The brain needs to engage in both these activities and switch between them flexibly. As we have discussed, there is evidence for both these modes of operation in the right IPL.

Importantly, neither of these processes are considered in several existing models of the visual system, including that of Milner and Goodale (Milner & Goodale, 1995; Mishkin et al., 1983; Rizzolatti & Matelli, 2003). Moreover, each of these modes of operation receives input from what might be termed ‘goal-directed’ as well as ‘bottom–up’ mechanisms. It therefore becomes difficult to view the functions of the SPL and IPL as goal-driven and stimulus-driven, respectively (Corbetta & Shulman, 2002), when both of these processes seem to rely so heavily on IPL activity.

We would rather conceptualise the IPL as contributing to two broadly different, but complementary, aspects of attention. As we hope to have shown, the evidence suggests it plays an important role in both responding to salient events as well as maintaining attention on the task at hand. The weight given to these processes appears to differ between the two hemispheres. This is most evident from a consideration of the two syndromes, hemineglect and limb apraxia, which result from damage to the right and left IPL, respectively (Buxbaum et al., 2007, 2005; De Renzi et al., 1980; Goldenburg, 1996; Haaland et al., 2000; Halsband et al., 2001; Heilman & Watson, 2001; Mort et al., 2003; Pazzaglia et al., 2008; Vallar & Perani, 1986). Neglect or disorders of attention following right hemisphere damage may be associated with deficits in sustaining attention and detecting salient events (Buxbaum et al., 2004; Friedrich et al., 1998; He et al., 2007; Hjaltason et al., 1996; Husain & Nachev, 2006; Husain & Rorden, 2003; Lhermitte et al., 1985; Robertson, 2001; Robertson et al., 1997; Rueckart & Grafman, 1998; Rueckert & Grafman, 1996), whereas there is no evidence of similar deficits encountered in patients with limb apraxia following left hemisphere lesions.

Of course, the defining feature of neglect is a difference in responding to stimuli in contralesional versus ipsilesional space. Such a spatial or directional impairment is not simply explained by a global deficit in sustaining attention or detecting salient events. Our argument is not that these two functions explain all of the neglect syndrome, or indeed all of IPL function, but rather that they may contribute to or exacerbate any spatial biases produced by unilateral lesions (for further discussion see Husain & Nachev, 2006 and Husain & Rorden, 2003). Spatial biases in attention may also follow left hemisphere lesions but they tend to be less pronounced and less persistent. We would hypothesize that the severity of neglect may be far less in such individuals than their right hemisphere counterparts because they do not also suffer from comparably severe deficits in sustaining attention or responding to salient items (measured with stimuli presented centrally). Such a proposal is obviously open to empirical testing in the future.

In summary, our review of current models of the visual system, suggest that the human IPL poses a real challenge to existing dichotomies. We argue that different aspects of IPL function are not adequately captured by these schemes. Our appraisal of imaging, electrophysiological and lesion findings regarding the IPL suggest that it plays a crucial role in flexibly reconfiguring behaviour between two states: maintaining attention on current task goals and responding to salient information and new events in the world around us.

## Figures and Tables

**Table 1:** Meta-analysis of studies assessing sustained attention. The studies included in this meta-analysis, employed tasks assessing vigilance/sustained attention, in any sensory modality, at *a single location* only in healthy control subjects. Only right hemisphere fronto-parietal activations are shown in this table and plotted in MRICro (Fig. 2).

Study	Task used	Modality of imaging	No of subjects	Regions activated	Talairach Coordinates (*x*, *y*, *z*)	*Z*/*t* score of activation^a^
Pardo et al. (1991)	Visual and somatosensory vigilance tasks	PET	23	Parietal lobe	29, −51, 34	>2.1
					35, −35, 48	>2.1
					39, −27, 46	>2.1
					49, −25, 46	>2.1
				Frontal lobe	45, 21, 34	>2.1
					31, 17, 44	>2.1
Johannsen et al. (1997)	Visual and vibration vigilance tasks	PET	17	IFG	44, 20, −8	3.1
				MFG	40, 34, 23	2.9
				IPL	43, −61, 44	3.5
Paus et al. (1997)	Auditory CPT	PET	8	VLPFC	36, 27, 12	3.6
					36, 22, −11	4.9
					38, 20, −5	3.7
				ILP	59, −37, 35	3.4
Coull and Frith (1998)^b^	Visual CPT variant	PET	4	IFG	36, 20, 10	4.59
				IPS	36, −56, 44	4.72
Coull et al. (1998)	Visual vigilance task	PET	6	IPL	48, −52, 36	4.42
				DLPFC	36, 10, 40	5.22
Hager et al. (1998)	Visual CPT	fMRI	12	DLPFC	30, 43, 44	5.44
					41, 38, 41	4.05
					37, 27, 33	2.71
Sturm et al. (1999)	Visual vigilance task	PET	15	MFG	36, 36, 32	4.74
					30, 46, 4	4.73
						
				IPL	54, −52, 24	4.29
Adler et al. (2001)	Visual CPT	fMRI	14	DLPFC	38, 43, 15.5	
				Anterior insula	34, 15, 11.5	
				IPL	38, −49, 39.5	
Vandenberghe et al. (2001)	Visual vigilance task	fMRI	12	Angular gyrus	54, −60, 33	6.44
					57, −51, 30	6.15
					60, −45, 42	5.26
				PMC	48, 12, 42	5.75
				MFG	39, 48, 21	5.27
				Anterior insula	51, 33, 21	5.24
					39, 30, −9	4.94
Foucher et al. (2004)	Visual vigilance task	fMRI	7	IFS	47.5, 41.1, 7.2	7.51
					39.6, 46, −10.7	4.95
				IPL	53.5, −40.1, 51.8	6.39
				SPL	33.7, −68.8, 49.5	6.36
Sturm et al. (2004)	Auditory vigilance task	PET	10	IFG	32, 26, −15	4.81
					40, 23, 3	4.63
				IPL	51, −49, 36	3.7

CPT, continuous performance task; IFG, inferior frontal gyrus; MFG, middle frontal gyrus; IPL, inferior parietal lobe; VLPFC, ventroleteral prefrontal cortex; DLPFC, dorsolateral prefrontal cortex; PMC, premotor cortex; IFS, inferior frontal sulcus.

a *Z*/*t* scores given where available.

b This study employed the same stimuli but different instructions in two tasks. In the sustained attention task subjects had to respond to all stimuli, in the selective attention task they had to respond to target stimuli only. The coordinates given here refer to areas activated by both tasks.

**Table 2:** Meta-analysis of studies assessing target-related salience detection. This meta-analysis included tasks performed at a *single location in space* only in healthy control subjects. Stimuli could be presented in any sensory modality. Only right hemisphere frontal and parietal activations are listed here and illustrated in Fig. 3.

Study	Modality of task	No of subjects	Regions activated	Talairach coordinates (*x*, *y*, *z*)	Z/t score of activation^a^
Linden et al. (1999)	Visual and auditory	5	IPL	52, −31, 41	
				55, −36, 35	
				41, −29, 48	
				55, −33, 31	
				55, −29, 26	
			IFL	48, 4, 11	
				44, 9, 9	
				42, 2, −3	
				43, 11, −4	
				45, 1, 44	
Clark et al. (2000)	Visual	6	IPL	48, −39.38	>3.09
			MFG	32, 0, 56	>3.09
			IFL	44, 17, 6	>3.09
Kiehl et al. (2001)	Visual and auditory	10	MFG	28, 48, 28	7.2
			IFG	52, 12, 28	6.67
			SPL	28, −56, 60	7.16
			IPL	60, −36, 24	9.42
Foucher et al. (2004)	Visual	7	MFG	27.7, 56.7, 6.4	4
			IPL	55.4, −58.8, 28.7	3.37
Huang et al. (2005)	Somatosensory	9	IPL	46.1, 46.6, 41.3	
			dPMA	36.6, −14, 54.8	
			DLPFC	37.5, 23.8, 30.4	
			VPMA	48.9, 6.1, 24.7	
Kiehl et al. (2005)	Auditory	100	MFG	23.8, 51.5, 19.5	16.69
			IFG	51.5, 8.5, 14.3	14.07
			Insula	43.6, 14.8, −14.2	18.34
			SPL	23.8, −47.4, 61.3	12.42
			IPL	55.4, −34, 20.1	21.25
Bunzeck and Duzel (2006)	Visual	14	Insula	33.7, 23.3, −1.2	9.3
				39.6, 0.8, 16.5	4.44
			MFG	39.6, 30.9, 35.3	5.88
			IPL	61.4, −22.1, 23.2	4.72
Lagopoulos et al. (2006)	Auditory	6	IPL	38, −52, 36	6.72
Gur et al. (2007)	Visual	36	IPL	52, −26, 44	4.57
			MFG	32, 50, 12	4.32
			Insula	40, −2, 16	4.38
Williams et al. (2007)	Auditory	16	IFG	59.4, 18.4, 17.5	4.14
			IPL	59.4, −41.2, 31.5	3.47

IPL, inferior parietal lobe; IFL, inferior frontal lobe; MFG, middle frontal gyrus; IFG, inferior frontal gyrus; SPL, superior parietal lobe; dPMA, dorsal premotor area; DLPFC, dorsolateral prefrontal cortex; VPMA, ventral premotor area.

a *Z*/*t* scores given where available.

**Table 3:** Meta-analysis of studies assessing orientation to novel distracters. Only right hemisphere activations within the frontal and parietal lobes are listed here. Investigations included all presented stimuli at a single location.

Study	Modality of task	No. of subjects	Regions activated	Talairach coordinates (*x*, *y*, *z*)	*Z*/*t* score of activation
Downar et al. (2002)^a^	Visual, auditory and somatosensory	10	TPJ/IPL	56, −36, 24	4.77
			IFG	53, 9, 26	4.34
				42, 0, 22	4.36
			Insula	43, 13, 4	4.27
Kiehl et al. (2001)	Visual and auditory	10	I/MFG	48, 4, 28	5.33
			IPL	32, −52, 56	6.51
			Precuneus	28, −76, 32	9.5
Kiehl et al. (2005)	Auditory	100	IFG	47.5, 16.8, 25	14.64
				47.5, 22.4, −7.9	13.91
			IPL	35.6, −60, 43.5	14.14
			SPL	47.5, −40.6, 42.6	11.87
Bunzeck and Duzel (2006)	Visual	14	Insula	29.7, 25.4, 2.4	4.67
			IFG	41.6, 13.1, 28.8	3.48
			MFG	45.5, 0, 38.7	3.69
Gur et al. (2007)	Visual	36	IFG	44, 6, 32	4.22

TPJ, temporoparietal junction; IPL, inferior parietal lobe; IFG, inferior frontal gyrus; MFG, middle frontal gyrus; SPL, superior frontal gyrus.

a Downer et al. (2002) did not use an oddball paradigm, but a similar task in which they were able to compare activity in response to novel stimuli to that obtained with a baseline familiar stimulus.

## Footnotes

1 It has previously been suggested on the basis of patient studies, that right IPL damage may be associated with difficulty in directing movements into the contralesional side of space – *directional hypokinesia* (Husain, Mattingley, Rorden, Kennard, & Driver, 2000; Mattingley, Husain, Rorden, Kennard, & Driver, 1998). However, lesions in those studies involved white matter as well as cortical regions. Thus, although maximal lesion overlap may have been in the IPL, fibres of passage from, for example, neighbouring structures such as the intraparietal sulcus (that are known to hold motor representations) might also have been involved.

2 We did not consider the more complex designs used by Coull and her colleagues, which used several different types of cue (Coull, Nobre, & Frith, 2001).

3 There are circumstances in which novel, or other infrequently occurring distractors, are capable of producing a P3a which is of larger amplitude than the P3b generated by the target in an oddball task (Combs & Polich, 2006; Comerchero & Polich, 1999; Katayama & Polich, 1998). These seem to be limited to situations in which the target is difficult to distinguish perceptually from the frequently occurring non-target stimuli, whilst the novels, or infrequently occurring distractors, are far more salient. In these instances however, although the amplitude of the P3b is reduced, the latency is increased. Also note that in such situations more errors occur, suggesting that subjects are less effectively engaged in the task.

## References

[bib1] Adler C.M., Sax K.W., Holland S.K., Schmithorst V., Rosenberg L., Strakowski S.M. (2001). Changes in neuronal activation with increasing attention demand in healthy volunteers: An fMRI study. Synapse.

[bib2] Arrington C.M., Carr T.H., Mayer A.R., Rao S.M. (2000). Neural mechanisms of visual attention: Object based selection of a region of space. Journal of Cognitive Neuroscience.

[bib3] Aston-Jones G., Cohen J.D. (2005). An integrative theory of locus coeruleus-norepinephrine function: Adaptive gain and optimal performance. Annual Reviews of Neuroscience.

[bib4] Aston-Jones G., Gonzalez M., Doran S., Ordway G.A., Schwartz M.A., Frazer A. (2007). Role of the locus coeruleus-norepinephrine system in arousal and circadian regulation of the sleep–wake cycle. Brain norepinephrine: Neurobiology and therapeutics.

[bib5] Aston-Jones G., Iba M., Clayton E., Rajkowski J., Cohen J., Ordway G.A., Schwartz M.A., Frazer A. (2007). The locus coeruleus and regulation of behavioral flexibility and attention: Clinical implications. Brain norepinephrine: Neurobiology and therapeutics.

[bib6] Aston-Jones G., Rajkowski J., Kubiak P., Alexinsky T. (1994). Locus coeruleus neurons in monkey are selectively activated by attended cues in a vigilance task. The Journal of Neuroscience.

[bib7] Aston-Jones, G., Rajkowski, J., Lu, W., Zhu, Y., Cohen, J. D., & Morecraft, R. J. (2002). Prominent projections from the orbital prefrontal cortex to the locus coeruleus in monkey. *Society for Neuroscience Abstract*, *28*, 86–89.

[bib8] Auerbach S.H., Alexander M.P. (1981). Pure agraphia and unilateral optic ataxia associated with a left superior parietal lobule lesion. Journal of Neurology, Neurosurgery and Psychiatry.

[bib9] Barcelo F., Suwazono S., Knight R.T. (2000). Prefrontal modulation of visual processing in humans. Nature Neuroscience.

[bib10] Berridge C.W., Waterhouse B.D. (2003). The locus coeruleus-noradrenergic system: Modulation of behavioral state and state-dependent cognitive processes. Brain Research. Brain Research Reviews.

[bib11] Bisiach E. (1993). Mental representation in unilateral neglect and related disorders: The twentieth Bartlett Memorial Lecture. The Quarterly Journal of Experimental Psychology A.

[bib12] Botvinick M.M., Cohen J.D., Carter C.S. (2004). Conflict monitoring and anterior cingulate cortex: An update. Trends in Cognitive Sciences.

[bib13] Brodmann, K. (1909). *Vergleichende Lokalisationslehre der Grosshirnrinde in ihren Prinzipien Dargerstelt auf Grund des Zellenbaues*. Leipzig.

[bib14] Buchsbaum B.R., Greer S., Chang W.L., Berman K.F. (2005). Meta-analysis of neuroimaging studies of the Wisconsin card-sorting task and component processes. Human Brain Mapping.

[bib15] Bunzeck N., Duzel E. (2006). Absolute coding of stimulus novelty in the human substantia nigra/VTA. Neuron.

[bib16] Butler S.H., Gilchrist I.D., Ludwig J.H., Muir K., Harvey M. (2006). Impairments of oculomotor control in a patient with a right temporo-parietal lesion. Cognitive Neuropsychology.

[bib17] Buxbaum L.J., Coslett H.B. (1998). Spatio-motor representations in reaching: Evidence for subtypes of optic ataxia. Cognitive Neuropsychology.

[bib18] Buxbaum L.J., Ferraro M.K., Veramonti T., Farne A., Whyte J., Ladavas E. (2004). Hemispatial neglect: Subtypes, neuroanatomy and disability. Neurology.

[bib19] Buxbaum L.J., Kyle K., Grossman M., Coslett H.B. (2007). Left inferior parietal representations for skilled hand-object interactions: Evidence from stroke and corticobasal degeneration. Cortex.

[bib20] Buxbaum L.J., Kyle K.M., Menon R. (2005). On beyond mirror neurons: Internal representations subserving imitation and recognition of skilled object-related actions in humans. Brain Research. Cognitive Brain Research.

[bib21] Clark V.P., Fannon S., Lai S., Benson R., Bauer L. (2000). Responses to rare visual target and distractor stimuli using event-related fMRI. Journal of Neurophysiology.

[bib22] Combs L.A., Polich J. (2006). P3a from auditory white noise stimuli. Clinical Neurophysiology.

[bib23] Comerchero M.D., Polich J. (1999). P3a and P3b from typical auditory and visual stimuli. Clinical Neurophysiology.

[bib24] Connolly J.D., Andersen R.A., Goodale M.A. (2003). FMRI evidence for a ‘parietal reach region’ in the human brain. Experimental Brain Research.

[bib25] Connolly J.D., Goodale M.A., DeSouza J.F.X., Menon R.S., Vilis T. (2000). A comparison of frontoparietal fMRI activation during anti-saccades and anti-pointing. Journal of Neurophysiology.

[bib26] Connolly J.D., Goodale M.A., Menon R.S., Munoz D.P. (2002). Human fMRI evidence for the neural correlates of preparatory set. Nature Neuroscience.

[bib27] Corbetta M., Kincade J.M., Ollinger J.M., McAvoy M.P., Shulman G.L. (2000). Voluntary orienting is dissociated from target detection in human posterior parietal cortex. Nature Neuroscience.

[bib28] Corbetta M., Shulman G.L. (2002). Control of goal-directed and stimulus-driven attention in the brain. Nature Reviews Neuroscience.

[bib29] Coull J.T., Frackowiak R.S., Frith C.D. (1998). Monitoring for target objects: Activation of right frontal and parietal cortices with increasing time on task. Neuropsychologia.

[bib30] Coull J.T., Frith C.D. (1998). Differential activation of right superior parietal cortex and intraparietal sulcus by spatial and non-spatial attention. Neuroimage.

[bib31] Coull J.T., Nobre A.C., Frith C.D. (2001). The noradrenergic alpha2 agonist clonidine modulates behavioural and neuroanatomical correlates of human attentional orienting and alerting. Cerebral Cortex.

[bib32] Coulthard E.J., Nachev P., Husain M. (2008). Control over conflict during movement preparation: Role of posterior parietal cortex. Neuron.

[bib33] Courchesne E., Hillyard S.A., Galambos R. (1975). Stimulus novelty, task relevance and the visual evoked potential in man. Electroencephalography and Clinical Neurophysiology.

[bib34] Culham J.C., Cavina-Pratesi C., Singhal A. (2006). The role of parietal cortex in visuomotor control: What have we learned from neuroimaging?. Neuropsychologia.

[bib35] Damasio A.R., Damasio H., Chang Chui H. (1980). Neglect following damage to frontal lobe or basal ganglia. Neuropsychologia.

[bib36] Dayan P., Yu A.J. (2006). Phasic norepinephrine: A neural interrupt signal for unexpected events. Network.

[bib37] De Renzi E., Motti F., Nichelli P. (1980). Imitating gestures: A quantitative approach to ideomotor apraxia. Archives of Neurology.

[bib38] Distler C., Boussaoud D., Desimone R., Ungerleider L.G. (1993). Cortical connections of inferior temporal area TEO in macaque monkeys. The Journal of Comparative Neurology.

[bib39] Downar J., Crawley A.P., Mikulis D.J., Davis K.D. (2000). A multimodal cortical network for the detection of changes in the sensory environment. Nature Neuroscience.

[bib40] Downar J.D., Crawley A.P., Mikulis D.J., Davis K.D. (2002). A cortical network sensitive to stimulus salience in a neutral behavioural context across multiple sensory modalities. Journal of Neurophysiology.

[bib41] Fan J., McCandliss B.D., Fossella J., Flombaum J.I., Posner M.I. (2005). The activation of attentional networks. NeuroImage.

[bib42] Farah M. (1995). Visual agnosia. Disorders of object recognition and what they tell us about normal vision.

[bib43] Foote S.L., Morrison J.H. (1987). Extrathalamic modulation of cortical function. Annual Reviews of Neuroscience.

[bib44] Foucher J.R., Otzenberger H., Gounot D. (2004). Where arousal meets attention: A simultaneous fMRI and EEG recording study. NeuroImage.

[bib45] Friedrich F.J., Egly R., Rafal R.D., Beck D. (1998). Spatial attention deficits in humans: A comparison of superior parietal and temporal-parietal junction lesions. Neuropsychology.

[bib46] Goldenburg G. (1996). Defective imitation of gestures in patients with damage in the left or right hemispheres. Neuropsychologia.

[bib47] Goodale M.A., Westwood D.A., Milner A.D. (2004). Two distinct modes of control for object-directed action. Progress in Brain Research.

[bib48] Gur R.C., Turetsky B.I., Loughead J., Waxman J., Snyder W., Ragland J.D. (2007). Haemodynamic responses in neural circuitries for detection of visual target and novelty: An event-related fMRI study. Human Brain Mapping.

[bib49] Haaland K.Y., Harrington D.L., Knight R.T. (2000). Neural representations of skilled movement. Brain.

[bib50] Hager F., Volz H.-P., Gaser C., Mentzel H.-J., Kaiser W.A., Sauer H. (1998). Challenging the anterior attentional system with a continuous performance task: A functional magnetic resonance imaging approach. European Archives of Psychiatry and Clinical Neuroscience.

[bib51] Hagmann P., Cammoun L., Gigandet X., Meuli R., Honey C.J., Wedeen V.J. (2008). Mapping the structural core of human cerebral cortex. PLoS Biology.

[bib52] Halsband U., Schmitt J., Weyers M., Binkofski F., Grutzner G., Freund H.J. (2001). Recognition and imitation of pantomimed motor acts after unilateral parietal and premotor lesions: A perspective on apraxia. Neuropsychologia.

[bib53] He B.J., Snyder A.Z., Vincent J.L., Epstein A., Shulman G.L., Corbetta M. (2007). Breakdown of functional connectivity in frontoparietal networks underlies behavioral deficits in spatial neglect. Neuron.

[bib54] Heilman K.M. (1992). Spatial dimensions in neglect. Journal of Clinical and Experimental Neuropsychology.

[bib55] Heilman K.M., Schwartz H.D., Watson R.T. (1978). Hypoarousal in patients with the neglect syndrome and emotional indifference. Neurology.

[bib56] Heilman K.M., Valenstein E., Watson R.T. (2000). Neglect and related disorders. Seminars in Neurology.

[bib57] Heilman K.M., Watson R.T., Heilman K.M., Valenstein E. (2001). Neglect and related disorders. Clinical Neuropsychology.

[bib58] Herrmann C.S., Knight R.T. (2001). Mechanisms of human attention: Event-related potentials and oscillations. Neuroscience and Biobehavioral Reviews.

[bib59] Hjaltason H., Tegner R., Tham K., Levander M., Ericson K. (1996). Sustained attention and awareness of disability in chronic neglect. Neuropsychologia.

[bib60] Hopfinger J.B., Buonocore M.H., Mangun G.R. (2000). The neural mechanisms of top–down attentional control. Nature Neuroscience.

[bib61] Howes D., Boller F. (1975). Simple reaction time: Evidence for focal impairments from lesions of the right hemisphere. Brain.

[bib62] Huang M.-X., Lee R.R., Miller G., Thoma R.J., Hanlon F.M., Paulson K.M. (2005). A parietal-frontal network studied by somatosensory oddball MEG responses, and its cross-modal consistency. NeuroImage.

[bib63] Husain M., Stein J.F. (1991). Visuospatial and visuomotor functions of the posterior parietal lobe. Vision and visual dysfunction (pp. . . . ): Vol 13, Dyslexia J. R. Cronly-Dillon (Series Ed).

[bib64] Husain M., Kennard C. (1996). Visual neglect associated with frontal lobe infarction. Journal of Neurology.

[bib65] Husain M., Mattingley J.B., Rorden C., Kennard C., Driver J. (2000). Distinguishing sensory and motor biases in parietal and frontal neglect. Brain.

[bib66] Husain M., Nachev P. (2006). Space and the parietal cortex. Trends in Cognitive Sciences.

[bib67] Husain M., Rorden C. (2003). Non-spatially lateralised mechanisms in hemispatial neglect. Nature Reviews Neuroscience.

[bib68] Ietswaart M., Carey D.P., Della Sala S., Dijkhuizen R.S. (2001). Memory-driven movements in limb apraxia: Is there evidence for impaired communication between the dorsal and the ventral streams?. Neuropsychologia.

[bib69] Jeannerod M. (1986). The formation of finger grip during prehension: A cortically mediated visuomotor pattern. Behavioural Brain Research.

[bib70] Jeannerod M., Decety J., Michel F. (1994). Impairment of grasping movements following bilateral posterior parietal lesion. Neuropsychologia.

[bib71] Johannsen P., Jakobsen J., Bruhn P., Hansen S.B., Gee A., Stodkilde-Jorgensen H. (1997). Cortical sites of sustained and divided attention in normal elderly humans. NeuroImage.

[bib72] Kastner S., Pinsk M.A., De Weerd P., Desimone R., Ungerleider L.G. (1999). Increased activity in human visual cortex during directed attention in the absence of visual stimulation. Neuron.

[bib73] Katayama J., Polich J. (1998). Stimulus context determines P3a and P3b. Psychophysiology.

[bib74] Kerkhoff G. (2001). Spatial hemineglect in humans. Progress in Neurobiology.

[bib75] Kiehl K.A., Laurens K.R., Duty T.L., Forster B.B., Liddle P.F. (2001). An event-related fMRI study of visual and auditory oddball tasks. Journal of Psychophysiology.

[bib76] Kiehl K.A., Stevens M.C., Laurens K.R., Pearlson G., Calhoun V.D., Liddle P.F. (2005). An adaptive reflexive processing model of neurocognitive function: Supporting evidence from a larger scale (*n* = 100) fMRI study of an auditory oddball task. NeuroImage.

[bib77] Knight R.T., Scabini D., Woods D.L., Clayworth C.C. (1989). Contributions of temporal-parietal junction to the human auditory P3. Brain Research.

[bib78] Lagopoulos J., Gordon E., Ward P. (2006). Differential BOLD responses to auditory target stimuli associated with a skin conductance response. Acta Neuropsychiatrica.

[bib79] Leclerq M., Leclerq M., Zimmerman P. (2002). Theoretical aspects of the main components and functions of attention. Applied neuropsychology of attention. Theory, diagnosis and rehabilitation.

[bib80] Lhermitte F., Turell E., LeBrigand D., Chain F. (1985). Unilateral visual neglect and wave P300. A study of nine cases with unilateral lesions of the parietal lobes. Archives of Neurology.

[bib81] Linden D.E.J., Prvulovic D., Formisano E., Vollinger M., Zanella F.E., Goebel R. (1999). The functional neuroanatomy of target detection: An fMRI study of visual and auditory oddball tasks. Cerebral Cortex.

[bib82] Liston C., Matalon S., Hare T.A., Davidson M.C., Casey B.J. (2006). Anterior cingulate and posterior parietal cortices are sensitive to dissociable forms of conflict in a task-switching paradigm. Neuron.

[bib83] Mackworth N.H. (1957). Some factors affecting vigilance. Advancements in Science.

[bib84] Malhotra P., Parton A., Greenwood R., Husain M. (2006). Noradrenergic modulation of space exploration in visual neglect. Annals of Neurology.

[bib85] Marois R., Leung H.-C., Gore J.C. (2000). A stimulus-driven approach to object identity and location processing in the human brain. Neuron.

[bib86] Mattingley J.B., Husain M., Rorden C., Kennard C., Driver J. (1998). Motor role of human inferior parietal lobe revealed in unilateral neglect patients. Nature.

[bib87] Mesulam M.M. (1999). Spatial attention and neglect: Parietal, frontal and cingulate contributions to the mental representation and attentional targeting of salient extrapersonal events. Philosophical Transactions of the Royal Society of London. Series B, Biological Science.

[bib88] Milner A.D. (1995). Cerebral correlates of visual awareness. Neuropsychologia.

[bib89] Milner A.D., Thier P., Karnath H.-O. (1997). Neglect, extinction, and the cortical streams of visual processing. Parietal lobe contributions to orientation in 3D space.

[bib90] Milner A.D. (1997). Vision without knowledge. Philosophical Transactions: Biological Science.

[bib91] Milner A.D., Goodale M.A. (1995). The visual brain in action.

[bib92] Milner A.D., Goodale M.A. (2008). Two visual systems re-viewed. Neuropsychologia.

[bib93] Milner A.D., Perrett D.I., Johnston R.S., Benson P.J., Jordan T.R., Heeley D.W. (1991). Perception and action in visual form agnosia. Brain.

[bib94] Mishkin M., Ungerleider L.G., Macko K.A. (1983). Object vision and spatial vision: Two cortical pathways. Trends in Neurosciences.

[bib95] Mort D.J., Malhotra M., Mannan S.K., Rorden C., Pambakian A., Kennard C. (2003). The anatomy of visual neglect. Brain.

[bib96] Nachev P., Husain M. (2006). Disorders of visual attention and the posterior parietal cortex. Cortex.

[bib97] Nachev P., Rees G., Parton A., Kennard C., Husain M. (2005). Volition and conflict in human medial frontal cortex. Current Biology.

[bib98] Nieuwenhuis S., Aston-Jones G., Cohen J.D. (2005). Decision making, the P3, and the locus coeruleus-norepinephrine system. Psychology Bulletin.

[bib99] Orban G.A., Claeys K., Nelissen K., Smans R., Sunaert S., Todd J.T. (2006). Mapping the parietal cortex of uman and non-human primates. Neuropsychologia.

[bib100] Orban G.A., Fize D., Peuskens H., Denys K., Nelissen K., Sunaert S. (2003). Similarities and differences in motion processing between the human and macaque brain: Evidence from fMRI. Neuropsychologia.

[bib101] Orban G.A., Van Essen D., Vanduffel W. (2004). Comparative mapping of higher visual areas in monkeys and humans. Trends in Cognitive Sciences.

[bib102] Pardo J.V., Fox P.T., Raichle M.E. (1991). Localization of a human system for sustained attention by positron emission tomography. Nature.

[bib103] Paus T., Zatorre R.J., Hofle N., Caramanos Z., Gotman J., Petrides M., Evans A.C. (1997). Time-related changes in neural systems underlying attention and arousal during the performance of an auditory vigilance task. Journal of Cognitive Neuroscience.

[bib104] Pazzaglia M., Smania N., Corato E., Aglioti S.M. (2008). Neural underpinnings of gesture discrimination in patients with limb apraxia. The Journal of Neuroscience.

[bib105] Perenin M.-T., Vighetto A. (1988). Optic ataxia: A specific disruption in visuomotor mechanisms. I. Different aspects of the deficit in reaching for objects. Brain.

[bib106] Perry R.J., Zeki S. (2000). The neurology of saccades and covert shifts in spatial attention: An event related fMRI study. Brain.

[bib107] Pineda J.A., Foote S.L., Neville H.J. (1989). Effects of locus coeruleus lesions on auditory, long-latency, event-related potentials in monkey. The Journal of Neuroscience.

[bib108] Pisella L., Binkofski F., Lasek K., Toni I., Rossetti Y. (2006). No double-dissociation between optic ataxia and visual agnosia: Multiple sub-streams for multiple visuo-manual integrations. Neuropsychologia.

[bib109] Polich J., Comerchero M.D. (2003). P3a from visual stimuli: Typicality, task, and topography. Brain Topography.

[bib110] Posner M.I., Boies S.J. (1971). Components of attention. Psychological Review.

[bib111] Posner M.I., Petersen S.E. (1990). The attention system of the human brain. Annual Reviews of Neuroscience.

[bib112] Posner M.I., Walker J.A., Friedrich F.J., Rafal R. (1984). Effects of parietal injury on covert orienting of attention. The Journal of Neuroscience.

[bib113] Rajkowski, J., Lu, W., Zhu, Y., Cohen, J.D., & Aston-Jones, G. (2000). Prominent projections from the anterior cingulate cortex to the locus coeruleus in Rhesus monkey. *Society for Neuroscience Abstract*, *26*, 838–845.

[bib114] Rajkowski J., Majczynski H., Clayton E., Aston-Jones G. (2004). Activation of monkey locus coeruleus neurons varies with difficulty and performance in a target detection task. Journal of Neurophysiology.

[bib115] Ritter W., Vaughan H.G., Costa L.D. (1968). Orienting and habituation to auditory stimuli: A study of short term changes in average evoked responses. Electroencephalography and Clinical Neurophysiology.

[bib116] Rizzolatti G., Matelli M. (2003). Two different streams form the dorsal visual system: Anatomy and functions. Experimental Brain Research.

[bib117] Robertson I. (2001). Do we need the “lateral” in unilateral neglect? Spatially nonselective attention deficits in unilateral neglect and their implications for rehabilitation. NeuroImage.

[bib118] Robertson I.H., Manly T., Beschin N., Daini R., Haeske-Dewick H., Homberg V. (1997). Auditory sustained attention is a marker of unilateral spatial neglect. Neuropsychologia.

[bib119] Robertson I.H., Mattingley J.B., Rorden C., Driver J. (1998). Phasic alerting of neglect patients overtcomes their spatial deficit in visual awareness. Nature.

[bib120] Rozzi S., Calzavara R., Belmalih A., Borra E., Gregoriou G.G., Matelli M. (2006). Cortical connections of the inferior parietal cortical convexity of the macaque monkey. Cerebral Cortex.

[bib121] Rueckart L., Grafman J. (1998). Sustained attention deficits in patients with lesions of posterior cortex. Neuropsychologia.

[bib122] Rueckert L., Grafman J. (1996). Sustained attention deficits in patients with right frontal lesions. Neuropsychologia.

[bib123] Rushworth M.F., Behrens T.E., Johansen-Berg H. (2006). Connection patterns distinguish 3 regions of human parietal cortex. Cerebral Cortex.

[bib124] Rushworth M.F., Buckley M.J., Behrens T.E., Walton M.E., Bannerman D.M. (2007). Functional organization of the medial frontal cortex. Current Opinion in Neurobiology.

[bib125] Rushworth M.F., Passingham R.E., Nobre A.C. (2005). Components of attentional set-switching. Experimental Psychology.

[bib126] Samuelsson H., Hjelmquist E., Jensen C., Ekholm S., Blomstrand C. (1998). Nonlateralised attentional deficits: An important component behind persisting visuospatial neglect?. Journal of Clinical and Experimental Neuropyschology.

[bib127] Santangelo V., Van der Lubbe R.H.J., Belardinelli M.O., Postma A. (2006). Spatial attention triggered by unimodal, crossmodal and bimodal exogenous cues: A comparison of reflexive orienting mechanisms. Experimental Brain Research.

[bib128] Schmahmann J.D., Pandya D.N., Wang R., Dai G., D’Arceuil H.E., de Crespigny A.J. (2007). Association fibre pathways of the brain: Parallel observations from diffusion spectrum imaging and autoradiography. Brain.

[bib129] Sereno M.I., Huang R.-S. (2006). A human parietal face area contains aligned head-centered visual and tactile maps. Nature Neuroscience.

[bib130] Shulman G.L., Ollinger J.M., Akbudak E., Conturo T.E., Snyder A.Z., Petersen S.E. (1999). Areas involved in encoding and applying directional expectations to moving objects. The Journal of Neuroscience.

[bib131] Smith D.B.D., Donchin E., Cohen L., Starr A. (1970). Auditory averaged evoked potentials in man during selective binaural listening. Electroencephalography and Clinical Neurophysiology.

[bib132] Squires N.K., Squires K.C., Hillyard S.A. (1975). Two varieties of long-latency positive waves evoked by unpredictable auditory stimuli in man. Electroencephalography and Clinical Neurophysiology.

[bib133] Stoet G., Snyder L.H. (2007). Correlates of stimulus–response congruence in the posterior parietal cortex. Journal of Cognitive Neuroscience.

[bib134] Sturm W., de Simone A., Krause B.J., Specht K., Hesselmann V., Radermacher I. (1999). Functional anatomy of intrinsic alertness: Evidence for a fronto-parietal-thalamic-brainstem network in the right hemisphere. Neuropsychologia.

[bib135] Sturm W., Longoni F., Fimm B., Dietrich T., Weis S., Kemma S. (2004). Network for auditory intrinsic alertness: A PET study. Neuropsychologia.

[bib136] Sturm W., Reul J., Willmes K. (1989). Is there a generalised right hemisphere dominance for mediating cerebral activation? Evidence from a choice reaction experiment with lateralised simple warning stimuli. Neuropsychologia.

[bib137] Sturm W., Thimm M., Kust J., Karbe H., Fink G.R. (2006). Alertness training in neglect: Behavioural and imaging results. Restorative Neurology and Neuroscience.

[bib138] Thiel C.M., Fink G.R. (2007). Visual and auditory alertness: Modality-specific and supramodal neural mechanisms and their modulation by nicotine. Journal of Neurophysiology.

[bib139] Thimm M., Fink G.R., Kust J., Karbe H., Sturm W. (2006). Impact of alertness training on spatial neglect: A behavioural and fMRI study. Neuropsychologia.

[bib140] Travers S., West R. (2008). Neural correlates of cue retrieval, task set reconfiguration, and rule mapping in the explicit cue task switching paradigm. Psychophysiology.

[bib141] Ungerleider L.G., Desimone R. (1986). Cortical connections of visual area MT in the macaque. The Journal of Comparative Neurology.

[bib142] Ungerleider L.G., Mishkin M., Ingle D.J., Goodale M.A., Mansfield R.J.W. (1982). Two cortical visual systems. Analysis of visual behavior.

[bib143] Usher M., Cohen J.D., Servan-Schreiber D., Rajkowski J., Aston-Jones G. (1999). The role of locus coeruleus in the regulation of cognitive performance. Science.

[bib144] Vallar G., Perani D. (1986). The anatomy of unilateral neglect after right-hemisphere stroke lesions. A clinical/CT-scan correlation study in man. Neuropsychologia.

[bib145] Vandenberghe R., Gitelman D.R., Parrish T.B., Mesulam M.M. (2001). Functional specificity of superior parietal medication of spatial shifting. NeuroImage.

[bib146] Vanduffel W., Fize D., Peuskens H., Denys K., Sunaert S., Todd J.T. (2002). Extracting 3D from motion: Differences in human and monkey intraparietal cortex. Science.

[bib147] Vaughan H.G., Ritter W. (1970). The sources of auditory responses recorded from the human scalp. Electroencephalography and Clinical Neurophysiology.

[bib148] Volpe U., Mucci A., Bucci P., Merlotti E., Galderisi S., Maj M. (2007). The cortical generators of P3a and P3b: A LORETA study. Brain Research Bulletin.

[bib149] Von Bonin G., Bailey P. (1947). The neocortex of *Macaca mulatta*.

[bib150] Von Economo C. (1929). The cytoarchitechtonics of the human cerebral cortex.

[bib151] Wager T.D., Jonides J., Reading S. (2004). Neuroimaging studies of shifting attention: A meta-analysis. Neuroimage.

[bib152] Wilkins A.J., Shallice T., McCarthy R. (1987). Frontal lesions and sustained attention. Neuropsychologia.

[bib153] Williams L.M., Felmingham K., Kemp A.H., Rennie C., Brown K.J., Bryant R.A. (2007). Mapping frontal-limbic correlates of orienting to change detection. NeuroReport.

[bib154] Wilson F.C., Manly T. (2003). Sustained attention training and errorless learning facilitates self-care functioning in chronic ipsilesional neglect following sever traumatic brain injury. Neuropsychological Rehabilitation.

[bib155] Wilson F.C., Manly T. (2003). Sustained attention training and errorless learning facilitates self-care functioning in chronic ipsilesional neglect following severe traumatic brain injury. Neuropsychological Rehabilitation.

[bib156] Yamaguchi S., Knight R.T. (1991). P300 generation by novel somatosensory stimuli. Electroencephalography and Clinical Neurophysiology.

[bib157] Yokoyama K., Jennings R., Ackles P., Hood P., Boller F. (1987). Lack of heart rate changes during an attention-demanding task after right hemisphere lesions. Neurology.

[bib158] Yu A.J., Dayan P. (2005). Uncertainty, neuromodulation, and attention. Neuron.

